# Choosing the Correct Guide Video on Central Venous Catheter Placement From YouTube

**DOI:** 10.7759/cureus.76102

**Published:** 2024-12-20

**Authors:** İlter Ağaçkıran, Merve Ağaçkıran

**Affiliations:** 1 Emergency Department, Hitit University Faculty of Medicine, Çorum, TUR; 2 Department of Emergency Medicine, Hitit University Erol Olçok Training and Research Hospital, Çorum, TUR

**Keywords:** central line placement, medicine education, quality, video analysis, youtube

## Abstract

Objective

Proper preparation is necessary before performing certain procedures on a patient. Recently, videos created using social media content have been used as a preparation method. This has become particularly important in medical education since the COVID-19 pandemic. Central venous catheter placement is an important procedure performed in critically ill patients. This study assessed the quality of videos about central venous catheter placement available on YouTube.

Methods

A search was conducted on YouTube with the keyword “central venous catheter placement” on June 1, 2024. The review process involved two independent medical experts who comprehensively assessed the relevance and content of each video. To assess the quality of the videos, the DISCERN score, mDISCERN score, Global Quality Score (GQS), and American Medical Association Journal (JAMA) rating scale were used as evaluation tools.

Results

Top 41 English-language videos deemed most relevant were evaluated, each with over 25,000 views. Analysis of the video sources revealed that 36.6% of the videos were uploaded by doctors. Moreover, 87.8% of the videos were audio narrations accompanied by real patients or mannequins, and the remaining videos comprised audio narration with animated images. The mean JAMA score of the videos was 1.73, the mean mDISCERN score was 2.66, the mean DISCERN score was 40.46, and the mean GQS score was 3.8. A statistically significant difference was observed between the mDISCERN scores of videos containing real visual content and those containing animated visual content (p = 0.038). Moreover, the same two groups also exhibited higher quality scores in the GQS (p = 0.029).

Conclusion

The quality of information related to central venous catheter placement on YouTube is highly variable. Specifically, videos that provide valuable information and those that have the potential to mislead viewers do not have a noticeable difference in terms of views and popularity. For a medical practitioner or physician seeking reliable information, a useful and safe approach is preferring videos uploaded by medical professionals. It is important to prioritize the professional identity of the content creator rather than the video’s popularity or the number of comments it has received.

## Introduction

Venous access is one of the most important and commonly performed invasive procedures for the care and monitoring of patients undergoing major surgery or on the intensive care unit [1]. Central venous access can be achieved through three routes: subclavian, jugular, and femoral. Although the jugular and subclavian routes are preferred over the femoral route owing to a higher risk of infection, they are more prone to complications compared to the femoral route [2,3]. Central venous access is utilized for indications such as parenteral nutrition, dialysis, plasmapheresis, medication administration, hemodynamic monitoring, and transvenous pacemaker placement [4-9].

Medical students and doctors use the internet for self-guided professional medical education, alongside their clinical teaching, education, and training [10,11]. In recent years, the internet has become a remarkable source of information, with social media platforms such as YouTube offering many educational videos [12]. Surgical assistants and medical students use these educational videos to prepare for surgery [13]. Gradually, the quality of content in YouTube videos has become a topic of debate due to inaccuracies or misleading information found in the published videos [14]. Specifically, the potential for misinformation in online videos has been frequently highlighted due to a lack of expertise and the absence of formal review processes among video producers [15-17].

Central venous catheter placement is vital for patients, and is a procedure that medical students and doctors need to thoroughly prepare for before performing it. This study aimed to assess the quality and reliability of YouTube videos related to central venous catheterization.

## Materials and methods

Data collection

The data used in the study were obtained from YouTube on July 1, 2024. The keyword “central venous catheter placement” was searched. Videos were ranked by the number of views. English videos about central venous catheter placement with more than 25,000 views were included in the study. Irrelevant content, videos containing personal experiences, repetitive content, non-English content, promotional videos, short films, and videos shorter than 60 seconds were excluded from the study. The data and upload sources of the videos were also recorded. The view rate, like rate, and video strength index (VPI) were also calculated using the data obtained from the videos. The view rate is obtained by dividing the number of views by the number of days since the video was uploaded. The like rate is obtained by multiplying the number of likes by 100 and dividing it by the sum of the number of likes and dislikes. The video strength index is obtained by multiplying the number of likes by the number of views by 100. All videos were evaluated by two independent emergency medicine specialists using the DISCERN, modified DISCERN (mDISCERN), Journal of the American Medical Association (JAMA) score, and Global Quality Score (GQS) rating systems. The average of these scores was calculated and recorded.

Evaluation criteria

The quality and reliability of the video content were assessed using the DISCERN score, mDISCERN score, JAMA score, and GQS rating. Two independent emergency medicine specialists evaluated the videos by watching them at different times. The average of the scores obtained from this evaluation was taken. Scores from both specialists were close to each other. The most comprehensive of the scoring systems used was the DISCERN scoring system, comprising two separate sets of 16 questions each that serve as collective evaluation criteria [18]. In the DISCERN scoring system, videos with scores between 16 and 26 represent a very low-quality standard, scores between 27 and 38 represent a low-quality standard, scores between 39 and 50 represent a moderate-quality standard, scores between 51 and 62 represent an acceptable-quality standard, and scores between 63 and 75 represent an excellent-quality standard [19,20]. The mDISCERN is a 5-point Likert scale adapted from a tool used to assess written health information. In this scale, scores are given as 0 or 1 based on criteria such as accuracy/clarity, reliability, balance, source, and uncertainty, with higher scores indicating greater reliability [21]. The JAMA score is also a quality assessment scale used to rate the accuracy and reliability of medical information on the internet. The scoring is based on four criteria: authorship, citation/references, explanation/conflict of interest, and validity, with a minimum score of 0 and a maximum score of 4 [22]. Lastly, GQS is a scoring system that subjectively evaluates the overall quality of video content on a scale from 1 to 5 [23].

In this study, publicly available YouTube videos were analyzed, and therefore, no human or animal subjects were involved. Therefore, ethical approval was not required [15,21].

Statistical analysis

Data were analyzed using IBM SPSS (version 23) (IBM Corp. Released 2015. IBM SPSS Statistics for Windows, Version 23.0. Armonk, NY: IBM Corp). Conformity to normal distribution was assessed with the Shapiro-Wilk test. Mann-Whitney U test was used to compare non-normally distributed data based on video content. Furthermore, the Kruskal-Wallis H test was used to compare non-normally distributed data based on video sources. The intraclass correlation coefficient (ICC) was used to determine the level of agreement between two independent observers. Spearman’s rank correlation coefficient was used to examine the relationships between non-normally distributed scores and video features. Results were presented as mean ± standard deviation and median (minimum-maximum) for quantitative data and frequency (percentage) for categorical data. Moreover, a *P*-value of <0.05 was considered statistically significant in all analyses.

## Results

The YouTube search yielded 41 English videos with more than 25,000 views. Analysis of the video sources revealed that 36.6% of the videos were uploaded by doctors. Moreover, 87.8% of the videos were audio narrations accompanied by real patients or mannequins, and the remaining videos was audio narration with animated images (Table 1). 

**Table 1:** Features of the videos

	n (%)
Source	
Independent user	7 (17.1)
Doctor	15 (36.6)
Hospital	5 (12.2)
Health professionals other than doctors	14 (34.1)
Content	
Animation	5 (12.2)
Real visual	36 (87.8)
Total	43

The average number of views for the videos was 275976.1 views. The average length of the videos was 555.37 seconds, average number of likes was 1980.39 likes, and average number of dislikes was 87.41 dislikes. Average number of comments on the videos was 42.88 comments, and the average time since upload was 2391.44 days. The mean JAMA score of the videos was 1.73, the mean mDISCERN score was 2.66, the mean DISCERN score was 40.46, and the mean GQS score was 3.8. The average VPI value of the videos was 221.33, the average like ratio was 228.36, and the average view ratio was 96.12 (Table 2). 

**Table 2:** Features of the videos Mean ± standard deviation, median (min–max), sec:second, JAMA: Journal of the American Medical Association Score, GQS: Global Quality Score, VPI:video power index

Variables		
Number of views	275976.1 ± 316800.04	143711 (27180-1327711)
Length of video(sec)	555.37 ± 314.88	545 (61-1560)
Likes	1980.39 ± 2836.6	1017 (81-14612)
Dislike	87.41 ± 124.25	26 (0-547)
Comments	42.88 ± 62.04	19.5 (0-341)
Time since upload	2391.44 ± 1652.24	1846 (182-5832)
JAMA	1.73 ± 0.95	2 (0-4)
Modified DISCERN	2.66 ± 1.04	3 (0-5)
DISCERN	40.46 ± 10.85	42 (16-55)
GQS	3.8 ± 1.19	4 (1-5)
VPI	221.33 ± 377.35	90.46 (6.65-1807.17)
Like ratio	228.36 ± 383.42	91.21 (6.65-1836.37)
View ratio	96.12 ± 3.21	96.52 (85.64-100)

The mDISCERN, JAMA, and GQS scores between the two observers were found to be in statistically good agreement (ICCJama: 0.912, ICCModifiedDiscern: 0.831, ICCGQS: 0.901, p < 0.001). A statistically significant difference was found in video length based on video content (p = 0.004). The median length of animated videos was 202 seconds, whereas the median length of videos with visual and audio content was 600.5 seconds. A statistically significant difference was found between the mDISCERN scores based on video content (p = 0.038). The median mDISCERN score for animated videos was 2, whereas the median score for videos with real patient content was 3. Furthermore, a statistically significant difference was found in GQS scores based on video content (p = 0.029). Moreover, the median GQS values of animated videos and those with real patient videos were 2 and 4, respectively. There was no statistically significant relationship between video content and other characteristics (p > 0.050) (Table 3). 

**Table 3:** Comparison of video features by content m: Mann–Whitney U test, mean ± standard deviation, median (min–max), JAMA: Journal of the American Medical Association Score, GQS: Global Quality Score, VPI:video power index

Variables	Content		Test statistic	p^m^
	Animation (n = 5)	Real visual (n = 35)		
Number of views	196988.8 ± 197110.51	286946.56 ± 330521.36	-0.159	0.873
	81509 (48746 - 510785)	156885 (27180 - 1327711)		
Length of video	209.8 ± 90.02	603.36 ± 304.89	-2.909	0.004
	202 (107 - 343)	600.5 (61 - 1560)		
Likes	715.2 ± 804.48	2156.11 ± 2976.81	-1.554	0.12
	297 (134 - 2073)	1068 (81 - 14612)		
Dislike	37 ± 60.32	94.42 ± 129.67	-1.236	0.216
	10 (0 - 144)	33.5 (0 - 547)		
Comments	20 ± 25.47	46.14 ± 65.19	-1.371	0.17
	8 (1 - 62)	21 (0 - 341)		
Time since upload	3573.2 ± 1982.84	2227.31 ± 1563.11	-1.394	0.163
	4136 (182 - 5246)	1782 (220 - 5832)		
JAMA	1.4 ± 1.14	1.78 ± 0.93	-0.783	0.434
	1 (0 - 3)	2 (0 - 4)		
Modified DISCERN	1.8 ± 0.84	2.78 ± 1.02	-2.08	0.038
	2 (1 - 3)	3 (0 - 5)		
GQS	2.6 ± 1.34	3.97 ± 1.08	-2.177	0.029
	2 (1 - 4)	4 (1 - 5)		
VPI	324.47 ± 648.59	207.01 ± 336.05	-1.116	0.265
	19.71 (9.82 - 1483.23)	90.77 (6.65 - 1807.17)		
Like ratio	329.26 ± 655.07	214.34 ± 342.52	-1.116	0.265
	19.71 (10.55 - 1499.4)	94.64 (6.65 - 1836.37)		
View ratio	96.32 ± 3.11	96.09 ± 3.27	-0.12	0.905
	96.09 (93.1 - 100)	96.81 (85.64 - 100)		

When content creators were examined, it was observed that the videos were most commonly uploaded by medical doctors (36.6%). However, videos with the highest average views and likes were those produced by healthcare professionals. When examining mean values of VPI and like ratios, content creators with the highest ratios were medical doctors. All sources had similar view ratios. No statistically significant difference was found between video features and scores according to sources (p > 0.050) (Table 4). 

**Table 4:** Comparison of video features according to sources h: Kruskal–Wallis H test, mean ± standard deviation, median (min–max),JAMA: Journal of the American Medical Association Score, GQS: Global Quality Score, VPI:video power index

	Source				Test statistic	p^h^
Variables	Independent user (n = 7)	Doctor (n = 15)	Hospital (n = 5)	Health professionals (n = 14)		
Number of views	147119.14 ± 153637.66	303072.2 ± 320555.43	120651.4 ± 128400.43	366846.14 ± 392708.76	2.883	0.41
	81509 (48746 - 484438)	192318 (27180 - 923674)	63984 (39157 - 345142)	219073.5 (30613 - 1327711)		
Length of video	464.29 ± 300.98	550.53 ± 277.69	498.8 ± 248.68	626.29 ± 387.36	0.823	0.844
	343 (107 - 929)	594 (61 - 948)	545 (197 - 829)	534.5 (155 - 1560)		
Likes	658.14 ± 561.82	2249.67 ± 2948.58	810 ± 415.39	2771 ± 3601.54	8.053	0.051
	305 (134 - 1616)	761 (81 - 8819)	663 (387 - 1384)	1871.5 (617 - 14612)		
Dislike	35.43 ± 33.42	106.2 ± 167.47	52.6 ± 100.42	105.71 ± 104.84	2.705	0.439
	22 (0 - 91)	32 (0 - 547)	12 (0 - 232)	98.5 (0 - 361)		
Comments	13 ± 12.57	43.71 ± 52.82	27 ± 22.96	62.64 ± 86.73	4.579	0.205
	14 (0 - 32)	18 (1 - 198)	18 (8 - 66)	32 (0 - 341)		
Time since upload	3783.14 ± 1023.15	2211.8 ± 1685.37	2368.2 ± 1294.01	1896.36 ± 1731.48	6.994	0.072
	4136 (1844 - 4787)	1720 (182 - 5027)	1913 (1193 - 4567)	1176.5 (519 - 5832)		
JAMA	1.57 ± 0.98	1.33 ± 0.82	2.4 ± 1.14	2 ± 0.88	6.6	0.086
	2 (0 - 3)	1 (0 - 3)	2 (1 - 4)	2 (1 - 4)		
Modified DISCERN	2.43 ± 1.27	2.33 ± 0.98	2.8 ± 0.84	3.07 ± 1	4.893	0.18
	2 (1 - 5)	2 (0 - 4)	3 (2 - 4)	3 (1 - 5)		
GQS	3.57 ± 0.98	3.67 ± 1.11	3.8 ± 1.1	4.07 ± 1.44	3.33	0.343
	4 (2 - 5)	4 (1 - 5)	4 (2 - 5)	5 (1 - 5)		
VPI	37.56 ± 33.45	373.46 ± 552.19	54.37 ± 57.21	209.84 ± 229.11	9.28	0.051
	22.92 (9.82 - 107.1)	185.04 (6.65 - 1807.17)	32.45 (14.01 - 154.52)	127.14 (35.79 - 904.19)		
Like ratio	39.43 ± 35.44	381.96 ± 559.62	59.82 ± 68.47	218.44 ± 235.77	8.985	0.051
	23.74 (10.55 - 113.13)	193.75 (6.65 - 1836.37)	32.82 (14.01 - 180.42)	137.6 (36.64 - 926.53)		
View ratio	95.64 ± 2.28	96.26 ± 2.79	96.31 ± 6	96.15 ± 3.12	1.562	0.668
	95.32 (93.1 - 100)	95.5 (91.3 - 100)	98.82 (85.64 - 100)	97.47 (90.69 - 100)		

A statistically significant moderately positive correlation was observed between the JAMA score and video length (r = 0.524, p < 0.001), the mDISCERN score and video length (r = 0.486, p = 0.001), the GQS and video length (r = 0.513, p = 0.001), and the mDISCERN score and number of likes (r = 0.449, p = 0.003). Furthermore, a statistically significant positive weak correlation was observed between GQS and number of likes (r = 0.348, p = 0.026), mDISCERN score and number of dislikes (r = 0.314, p = 0.045), the mDISCERN score and number of comments (r = 0.358, p = 0.023), GQS and number of comments (r = 0.324, p = 0.041), the mDISCERN score and VPI (r = 0.368, p = 0.018), and the mDISCERN score and like ratio (r = 0.368, p = 0.018). No statistically significant relationships were observed between the scores and other characteristics (p > 0.050) (Table 5). 

**Table 5:** Investigating the relationship between scores and video features r: Spearman’s rho correlation coefficient, JAMA: Journal of the American Medical Association Score, GQS: Global Quality Score, VPI:video power index

Variables		JAMA	Modified DISCERN	GQS
Number of Views	r	-0.061	0.294	0.07
	p	0.706	0.062	0.663
Length of video	r	0.524	0.486	0.513
	p	<0.001	0.001	0.001
Likes	r	0.146	0.449	0.348
	p	0.364	0.003	0.026
Dislike	r	0.132	0.314	0.198
	p	0.411	0.045	0.214
Comments	r	0.132	0.358	0.324
	p	0.415	0.023	0.041
Time since upload	r	-0.184	-0.155	-0.273
	p	0.25	0.332	0.085
VPI	r	0.073	0.368	0.219
	p	0.651	0.018	0.168
Like ratio	r	0.081	0.368	0.214
	p	0.613	0.018	0.18
View ratio	r	0.007	0.034	0.137
	p	0.967	0.835	0.391

## Discussion

YouTube is the world’s largest video-sharing platform and has recently been providing free informative videos on health topics for doctors and patients. However, the videos uploaded on YouTube are often not reviewed by experts in the field and lack reliability [19,24,25]. Moreover, a review of the relevant literature revealed that studies that assess the quality and reliability of YouTube videos related to central venous catheter placement are rare. The overall quality and reliability scores of the videos in this study were at a moderate level.

This study emphasizes that videos on central venous catheter placement with animated visuals and voice-over are of lower quality compared to those with real images. Such a distinction has not been previously made in the literature. Based on the mDISCERN and GQS evaluation of the publicly available videos on central venous catheter insertion in this study, videos with real content were deemed to be significantly superior. Animated videos had lower engagement compared to other videos. The primary reason for the lack of engagement with animated videos may be due to their low quality. So et al. demonstrated that, despite the video quality not being the best, the most-liked videos were animated [26]. In the study by Satılmış and Başaran, one of the shortcomings was the lack of emphasis on information aimed at reducing potential complications during the procedure [27]. The number of videos addressing complications was also low as in the present study.

The present study did not reveal significant differences in reliability and quality between hospitals, doctors, healthcare professionals, and independent users. This finding was consistent with the literature [28]. In the present study, it was found that content created by healthcare professionals received higher scores according to the GQS and mDISCERN classifications. The reason for these producers creating higher quality videos is believed to be due to the collaboration of multiple individuals in content creation.

A strength of this study was the independent scoring of the videos by two separate experts. Our study has some limitations. One of the limitations is that only the YouTube platform was used, other social media platforms were not used. This limits the generalizability of the findings, but YouTube is the most used video-sharing platform among people. Another limitation is that the number of views of videos on YouTube changes every day, which is due to the current structure of YouTube. Therefore, it should be known that video analyses based on these platforms may change over time. Limitations of this study include the inclusion of only English videos and the selection of videos with over 25,000 views. Therefore, the small number of samples (n=41) is an important limitation. We recommend conducting new studies with larger sample sizes.

## Conclusions

In the present study, four different quality scales indicated that YouTube videos related to central venous catheter placement contained information of medium quality. Although incorrect demonstration of the technique was rare, several videos lacked information on potential complications during the procedure and failed to cite the sources of the information provided. Some videos with high engagement were of low quality. Thus, it was deemed that watching videos created by healthcare professionals would be a better choice for obtaining reliable information.
